# Restenosis rates in patients with ipsilateral carotid endarterectomy and contralateral carotid artery stenting

**DOI:** 10.1371/journal.pone.0262735

**Published:** 2022-02-11

**Authors:** Dat Tin Nguyen, Boldizsár Vokó, Balázs Bence Nyárádi, Tamás Munkácsi, Ákos Bérczi, Zoltán Vokó, Edit Dósa

**Affiliations:** 1 Heart and Vascular Center, Semmelweis University, Budapest, Hungary; 2 Hungarian Vascular Radiology Research Group, Budapest, Hungary; 3 Center for Health Technology Assessment, Semmelweis University, Budapest, Hungary; Medical University Innsbruck, AUSTRIA

## Abstract

**Purpose:**

We aimed to evaluate the long-term outcome of carotid endarterectomy (CEA) and carotid artery stenting (CAS) in patients who underwent both procedures on different sides.

**Methods:**

In this single-center retrospective study (2001–2019), 117 patients (men, N = 78; median age at CEA, 64.4 [interquartile range {IQR}, 57.8–72.2] years; median age at CAS, 68.8 [IQR, 61.0–76.0] years) with ≥50% internal carotid artery stenosis who had CEA on one side and CAS on the other side were included. The risk of restenosis was estimated by treatment adjusted for patient and lesion characteristics.

**Results:**

Neurological symptoms were significantly more common (41.9% vs 16.2%, P<0.001) and patients had a significantly shorter mean duration of smoking (30.2 [standard deviation {SD}, 22.2] years vs 31.8 [SD, 23.4] years, P<0.001), hypertension (10.1 [SD, 9.8] years vs 13.4 [SD, 9.1] years, P<0.001), hyperlipidemia (3.6 [SD, 6.6] years vs 5.0 [SD, 7.3] years, P = 0.001), and diabetes mellitus (3.9 [SD, 6.9] years vs 5.7 [SD, 8.9] years, P<0.001) before CEA compared to those before CAS. While the prevalence of heavily calcified stenoses on the operated side (25.6% vs 6.8%, P<0.001), the incidence of predominantly echogenic/echogenic plaques (53.0% vs 70.1%, P = 0.011) and suprabulbar lesions (1.7% vs 22.2%, P<0.001) on the stented side was significantly higher. Restenosis rates were 10.4% at 1 year, 22.3% at 5 years, and 33.7% at the end of the follow-up (at 11 years) for CEA, while these were 11.4%, 14.7%, and 17.2%, respectively, for CAS. Cox regression analysis revealed a significantly higher risk of restenosis (hazard ratio [HR], 1.80; 95% confidence interval [CI], 1.05–3.10; P = 0.030) for CEA compared to that for CAS. After adjusting for relevant confounding factors (smoking, hypertension, diabetes mellitus, calcification severity, plaque echogenicity, and lesion location), the estimate effect size materially did not change, although it did not remain statistically significant (HR, 1.85; 95% CI, 0.95–3.60; P = 0.070).

**Conclusion:**

Intra-patient comparison of CEA and CAS in terms of restenosis tilts the balance toward CAS.

## Introduction

Among invasive interventions, those performed for carotid artery stenosis are one of the most common in the field of vascular medicine [1]. To date, carotid artery stenting (CAS) still occurs primarily when the patient is at “high” risk for open surgery [2]. Patients with high or low placed stenosis, non-atherosclerotic lesions, contralateral carotid artery occlusion, contralateral laryngeal nerve palsy, previous radical neck surgery, cervical radiation therapy, and/or severe cardiopulmonary comorbidities are considered to be at “high” risk for open surgery [2, 3]. In all other cases, carotid endarterectomy (CEA) is preferable, especially if the patient has crescendo symptoms, aortic arch anomaly, aortic arch atheroma, tortuous common carotid artery (CCA), angulated internal carotid artery (ICA) origin, angulated distal ICA, severely diseased CCA/external carotid artery, pinhole or long ICA stenosis, and if the ICA lesions are thrombotic, heavily calcified, or sequential [2, 4, 5]. Age requires special attention when choosing the type of invasive therapy; the outcome of older patients is better with CEA than with CAS [6, 7]. The dominance of CEA over CAS is mainly because the perioperative death and stroke rate of CEA is lower than that of CAS [2, 6–13]. A meta-analysis of data from four randomized clinical trials (RCTs) revealed a 30-day death/stroke rate of 1.6% after CEA versus 2.7% after CAS in asymptomatic patients at “average” risk for surgery [2, 8–11]. The Endarterectomy versus Stenting in Patients with Symptomatic Severe Carotid Stenosis (EVA-3S) study, the Stent-Protected Angioplasty versus Carotid Endarterectomy (SPACE) trial, the International Carotid Stenting Study (ICSS), and the Carotid Revascularization Endarterectomy Versus Stenting Trial (CREST) have reported even stronger trends in symptomatic patients [2, 6, 7, 12, 13].

According to the results of large RCTs, it can be clearly stated that, among the long-term outcomes, there is no significant difference between CEA and CAS in the incidence of ipsilateral stroke [2, 6, 7, 12, 13]. However, concerning restenosis, the literature data are rather contradictory; while RCTs have shown that CAS has a higher rate of restenosis [2, 14, 15], observational studies have noted that CEA has a higher rate of restenosis [16–18].

Because there are two ICAs, it is possible to evaluate the short- and long-term outcomes of CEA and CAS in the same patient (ipsilateral CEA versus contralateral CAS). As the number of such publications is few (we found four in total) with small sample sizes (63 subjects the most) [19–22], we considered it worthwhile to perform an intra-patient comparison of restenosis on a larger sample size.

## Patients and methods

### Study characteristics

In this single-center retrospective study, 117 patients who underwent CEA on one side and CAS on the other side between January 2001 and January 2019 were included. The study was approved by the institutional ethics committee (Approval No: 222/2017). Due to the retrospective nature of the study, patient informed consent for analysis of data was not obtained. All data were fully anonymized before we accessed them.

### Procedure characteristics

The indications for CEA and CAS were based on international guidelines that were in force at the time of the intervention [23–25]. In general, asymptomatic patients in the presence of ≥70% ICA stenosis and symptomatic patients in the presence of ≥50% ICA stenosis were treated invasively. Following the guidelines, a team of vascular surgeons, interventional radiologists, and angiologists decided on the type of invasive therapy [23–25].

The type of open surgery was eversion endarterectomy (EEA) without routine shunt use, which was carried out under general anesthesia. The indication for the use of shunt was left to the discretion of the operating surgeon. The technical details of EEA can be found in the publication by Hirschberg et al. [16]. If the patient had not previously taken a platelet aggregation inhibitor drug regularly, antiplatelet therapy was initiated at least 7 days before the procedure and was continued indefinitely postsurgically. Technical success was defined as the absence of visible plaque remnants and successful restoration of blood flow at the completion of EEA [26].

CAS, which meant the deployment of a self-expanding stent, was executed in a standard manner via the common femoral, brachial, or radial artery. Embolic protection systems were routinely used and stent postdilation was inevitable. Puncture sites were manually compressed or sealed with a closure device. If they were not already administered, patients were pretreated with dual antiplatelet therapy for 5 days before the intervention and were administered postprocedurally for up to 3 months. If the patient did not have heart disease, monotherapy was sufficient thereafter. Technical success was achieved if no extravasation, dissection, or >30% residual stenosis was seen on the final angiographic images [23–27].

### Control ultrasonography

The carotid ultrasonography was due 6 weeks, 6 months, and 12 months after the procedure, and then once a year. In the event of symptom/complaint or contralateral invasive treatment, these dates were changed. On the operated side, a restenosis was considered 50–69% when the peak systolic velocity (PSV) was 210–270 cm/s and ≥70% when the PSV was >270 cm/s [28], while on the stented side, a restenosis was considered 50–69% when the PSV was 225–350 cm/s and ≥70% when the PSV was >350 cm/s [29].

### Evaluated parameters and their definitions

Symptoms were categorized as amaurosis fugax, transient ischemic attack (TIA), minor stroke, major stroke, or no symptoms [26].

Information on the presence and duration of atherosclerotic risk factors and the type and duration of medications was obtained from the medical record archiving system (MedSol; T-Systems Hungary Ltd., Budapest, Hungary) or directly from the patients, who were called by phone. Definitions of atherosclerotic risk factors can be found in one of the articles by Vértes et al. [30].

Stenosis grade, lesion length, and the severity of plaque calcification were evaluated on the reformatted computed tomography (CT)/CT angiography images. The percentage of ICA stenosis was determined according to the North American Symptomatic Carotid Endarterectomy Trial (NASCET) criteria [31]. Lesion length was defined as the distance between the proximal and the distal point where the degree of stenosis decreased to 80% of its maximum [32]. In terms of calcification, the following groups were distinguished: absent, mild (thin, discontinuous), moderate (thin, continuous or thick, discontinuous), and severe (thick, continuous) [33]. Plaque echogenicity was assessed by computer-assisted quantification of the gray-scale median (GSM) values using Adobe Photoshop software (CS3; Adobe System, San Jose, CA, USA) [34]. According to the modified Geroulakos classification, depending on the percentage of pixels in the plaque area with GSM values >25, plaques were categorized as type 1: uniformly echolucent (<15%), type 2: predominantly echolucent (15–50%), type 3: predominantly echogenic (50–85%), type 4: uniformly echogenic (>85%), and type 5: indeterminable due to acoustic shadow [35].

### Statistical analysis

Statistical analysis was performed with the software Stata 16.0 (StataCorp. 2019. Stata Statistical Software: Release 16. College Station, TX: StataCorp LLC). Distributions of the continuous variables in the two treatment groups were compared with paired t-tests, while categorical data were compared with Fisher exact tests. The probability of being free from restenosis was estimated by the Kaplan-Meier method. Restenosis was considered as failure in the analysis, while death and end of observation were considered as censoring. As only one death occurred during the 11-year follow-up, a competing risk problem was not considered. The maximum follow-up time was set at 11 years on each side. The event-free survival curves were compared by the log-rank test.

The crude hazard ratio (HR) comparing the two procedures was estimated by Cox proportional-hazards model. Then, we adjusted for prognostic factors that could be related to the indication of the procedures in a stepwise manner. First, we considered age, symptoms, and lesion characteristics, which were significantly associated with the procedures at the level of P≤0.20 in the univariate analysis. To improve the efficiency of the estimation, we eliminated the potential confounders of treatment effect in a stepwise manner from the initial model starting with the ones having the largest P-value in the Wald-test. The decision rule of elimination was that covariates non-significant at the level of P<0.05 were eliminated if the estimate of the coefficient of the treatment effect from the initial model did not change more than 10% by the elimination. After this process, lesion characteristics that were not previously included in the model were added back individually and were kept in the model if the adjustment for them changed the coefficient of treatment more than 10%. Finally, the length of history of smoking, hypertension, hyperlipidemia, and diabetes mellitus were added to the model, and the same elimination process was performed.

## Results

### Patient data

There were 39 women and 78 men in the study group (median age at CEA, 64.4 [interquartile range {IQR}, 57.8–72.2] years; median age at CAS, 68.8 [IQR, 61.0–76.0] years). Neurological symptoms were significantly more common (P<0.001) before CEA compared to those before CAS. Except for age ≥80 years (P = 0.033), there was no significant difference in the presence of atherosclerotic risk factors/comorbidities at the time of CEA and CAS. The duration of smoking (P<0.001), hypertension (P<0.001), hyperlipidemia (P = 0.001), and diabetes mellitus (P<0.001) was significantly shorter at the time of CEA compared to that at the time of CAS. (Table 1)

**Table 1:** Preprocedural symptoms and the presence and duration of atherosclerotic risk factors/comorbidities at the time of intervention.

Preprocedural symptoms and atherosclerotic risk factors/comorbidities	CEA (N = 117)	CAS (N = 117)	P-value
Preprocedural symptoms, N (%)	49 (41.9)	19 (16.2)	<0.001
Amaurosis fugax, N (%)	3 (2.6)	2 (1.7)	1.000
TIA, N (%)	27 (23.1)	9 (7.7)	0.002
Minor stroke, N (%)	11 (9.4)	6 (5.1)	0.314
Major stroke, N (%)	8 (6.8)	2 (1.7)	0.102
Presence of atherosclerotic risk factors/comorbidities			
Age ≥80 years, N (%)	5 (4.3)	15 (12.8)	0.033
BMI ≥30 kg/m^2^, N (%)	18 (15.4)	13 (11.1)	0.335
Smoking, N (%)	80 (68.4)	65 (55.6)	0.059
Hypertension, N (%)	108 (92.3)	109 (93.2)	1.000
Hyperlipidemia, N (%)	58 (49.6)	56 (47.9)	1.000
Diabetes mellitus, N (%)	44 (37.6)	45 (38.5)	0.896
Chronic kidney disease, N (%)	3 (2.6)	6 (5.1)	0.499
Duration of atherosclerotic risk factors/comorbidities			
*Smoking (years), mean (SD)	30.2 (22.2)	31.8 (23.4)	<0.001
†Hypertension (years), mean (SD)	10.1 (9.8)	13.4 (9.1)	<0.001
†Hyperlipidemia (years), mean (SD)	3.6 (6.6)	5.0 (7.3)	0.001
†Diabetes mellitus (years), mean (SD)	3.9 (6.9)	5.7 (8.9)	<0.001
†Chronic kidney disease (years), mean (SD)	0.1 (1.2)	0.2 (1.7)	0.107

*Duration: the aggregate period being an active smoker before the intervention.

†Duration: the period between the detection of hypertension, hyperlipidemia, diabetes mellitus or chronic kidney disease and the intervention.

*BMI*, Body mass index; *CAS*, carotid artery stenting; *CEA*, carotid endarterectomy; *SD*, standard deviation; *TIA*, transient ischemic attack.

### Lesion characteristics

Except for one stented stenosis (0.9%), which was presumably caused by radiation therapy, all other lesions (99.1%) were of atherosclerotic origin. There was no difference in the grade and length of the stenosis, but the majority of the lesions (79.5%) were mildly or moderately calcified in the CAS group compared to the balanced distribution of the lesions along the four categories of calcification in the CEA group. In terms of plaque echogenicity, most of the lesions were types 3 and 4 in both groups; however, significantly more lesions were types 3 and 4 in the CAS group compared to those in the CEA group. The majority of the lesions were in the bulb in both groups; suprabulbar lesions were mostly treated with stenting. (Table 2)

**Table 2:** Lesion characteristics.

Characteristics/parameters	CEA (N = 117)	CAS (N = 117)	P-value
Etiology			
Atherosclerosis, N (%)	117 (100)	116 (99.1)	1.000
Radiation-induced arteriopathy, N (%)	0 (0)	1 (0.9)	1.000
Stenosis grade (%), mean (SD)	85.9 (6.0)	85.8 (6.2)	1.000
Lesion length (mm), mean (SD)	10.5 (4.9)	10.2 (4.6)	0.523
Calcification			
Mild, N (%)	28 (23.9)	63 (53.8)	<0.001
Moderate, N (%)	29 (24.8)	30 (25.6)	1.000
Heavy, N (%)	30 (25.6)	8 (6.8)	<0.001
Echogenicity			
Types 1 and 2, N (%)	20 (17.1)	10 (8.5)	0.077
Types 3 and 4, N (%)	62 (53.0)	82 (70.1)	0.011
Type 5, N (%)	35 (29.9)	25 (21.4)	0.178
Location			
Bifurcation, N (%)	23 (19.7)	16 (13.7)	0.293
Bulb, N (%)	92 (78.6)	75 (64.1)	0.020
Suprabulbar segment, N (%)	2 (1.7)	26 (22.2)	<0.001

*CAS*, Carotid artery stenting; *CEA*, carotid endarterectomy; *SD*, standard deviation.

### Procedures

In 95 patients, CEA was the first invasive therapeutic method. The median time interval between the two procedures was 50.0 (IQR, 8.5–102.0) months if the first procedure was CEA and 2.5 (IQR, 1.0–12.8) months if the first procedure was CAS.

During CEA, the clamping time was 22.7±8.0 minutes. A shunt was inserted in four patients (3.4%).

In the case of CAS, access sites were femoral in 77 patients (65.8%), radial in 37 patients (31.6%), and brachial in three patients (2.6%). In two cases (1.7%), a proximal Mo.Ma (Medtronic Inc., Minneapolis, MN, USA) was used, while in the other patients (98.3%), a distal embolic protection device (FilterWire EZ; Boston Scientific Corp., Marlborough, MA, USA) was used. Predilation was carried out in six cases (5.1%). Balloon and stent parameters can be found in Table 3.

**Table 3:** Parameters of the balloons and stents.

Balloons/stents	Manufacturer	Size (mm), diameter (min–max) x length (min–max)
Balloons used for predilation (N = 6)		
Sterling (N = 3)	Boston Scientific Corp., Marlborough, MA, USA	3–4 x 15–25
Trek Rx (N = 1)	Abbott Vascular Inc., Santa Clara, CA, USA	3.5 x 25
Emerge (N = 1)	Boston Scientific Corp., Marlborough, MA, USA	3.5 x 20
Pantera Pro (N = 1)	Biotronik AG, Bülach, Switzerland	3 x 25
Stents (N = 117)		
Wallstent (N = 102)	Boston Scientific Corp., Marlborough, MA, USA	7–9 x 30–50
Exact (N = 7)	Abbott Vascular Inc., Santa Clara, CA, USA	7–9 x 30–40
Precise Pro (N = 6)	Cordis Corp., Johnson & Johnson Co., Miami, FL, USA	7–8 x 30–40
Cristallo Ideale (N = 1)	Invatec S.p.A., Roncadelle, Italy	7 x 40
Roadsaver (N = 1)	Terumo Corp., Tokyo, Japan	7 x 30
Balloons used for postdilation (N = 117)		
Sterling (N = 53)	Boston Scientific Corp., Marlborough, MA, USA	4–6 x 20–40
Maverick (N = 32)	Boston Scientific Corp., Marlborough, MA, USA	4–6 x 20
Ultra-Soft SV (N = 21)	Boston Scientific Corp., Marlborough, MA, USA	4–6 x 20–25
Rx Viatrac 14 Plus (N = 11)	Abbott Vascular Inc., Santa Clara, CA, USA	4–6 x 20–40

The technical success rate among the patients was 100%.

### Early (≤30 days) postprocedural period

No one died during the early stages of the follow-up.

The following complications occurred after CEA: four wound hematomas (3.4%), with two requiring surgical evacuation; five cranial nerve injuries (4.3%); two cases of hemodynamic instability (1.7%; hypotension, N = 1; hypertension, N = 1); one myocardial infarction (0.9%); and five neurological ischemic events (4.3%; TIA, N = 1; ipsilateral minor stroke, N = 1; ipsilateral major stroke, N = 3). Two of five neurological ischemic events were due to acute occlusion of the ICA that was operated on; both patients underwent reoperation.

The following complications were observed after CAS: one postpuncture pseudoaneurysm (0.9%), which was eliminated by thrombin injection; four cases of hemodynamic instability (3.4%; hypotension, N = 3; hypertension, N = 1); and six ocular or neurological ischemic events (5.1%; amaurosis fugax, N = 1; TIA, N = 5). One of six neurological ischemic events was due to acute stent occlusion; the patient underwent surgical stent removal.

There was no significant difference (P = 0.683 and P = 1.000, respectively) between CEA and CAS neither for hemodynamic instability nor for early postprocedural neurological complications.

### Follow-up

The median follow-up time was 10.0 (IQR, 5.5–14.0) years after CEA and 6.0 (IQR, 3.0–10.0) years after CAS. One death occurred during the follow-up period. The cause of death was ventricular fibrillation.

Ocular or neurological ischemic events corresponding to the operated side were reported in five patients (4.3%; amaurosis fugax, N = 1; TIA, N = 2; minor stroke, N = 2) and neurological symptoms corresponding to the stented side in one patient (0.9%; TIA, N = 1); the difference between the two sides was statistically non-significant (P = 0.213).

After CEA, 50–69% restenosis was detected in eight cases (6.8%; symptomatic restenosis, N = 0), 70–99% restenosis in 30 cases (25.6%; symptomatic restenosis, N = 5), and occlusion in two cases (1.7%; symptomatic occlusion, N = 0). Twenty-four of 30 patients with 70–99% restenosis underwent radiological intervention (Wallstent implantation [N = 24]; Boston Scientific Corp., Marlborough, MA, USA), while all the other patients remained on best medical treatment (BMT). Three patients (2.6%; symptomatic re-restenosis, N = 1) developed 70–99% re-restenosis; radiological reintervention (percutaneous transluminal angioplasty [PTA]) was performed in one of these three patients.

After CAS, 50–69% restenosis was detected in 12 cases (10.3%; symptomatic restenosis, N = 0), 70–99% restenosis in five cases (4.3%; symptomatic restenosis, N = 0), and occlusion in one case (0.9%; symptomatic occlusion, N = 1). Four of five patients with 70–99% restenosis underwent radiological reintervention (PTA), while all the others remained on BMT. No one had 70–99% re-restenosis.

Nine patients developed restenosis on both sides, with one patient having bilateral ICA occlusion.

**Fig 1:**
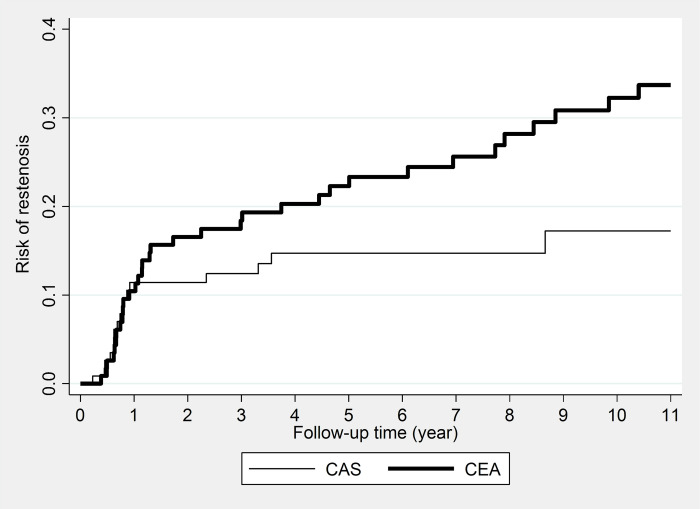
Risk of restenosis by treatment group. *CAS*, Carotid artery stenting; *CEA*, carotid endarterectomy.

**Fig 2:**
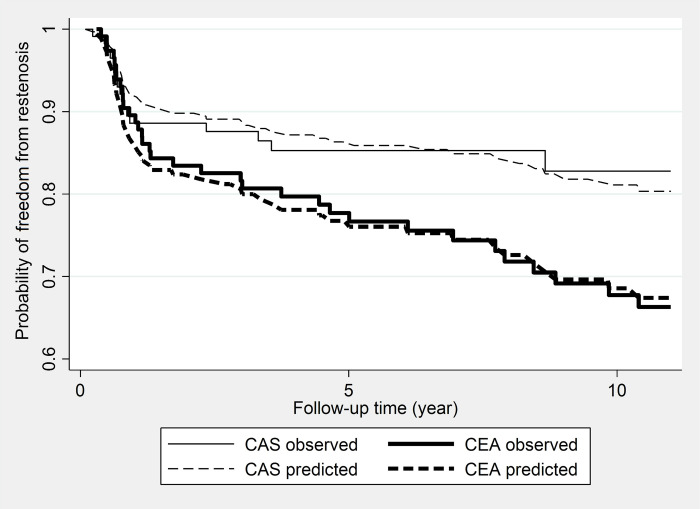
Observed and model predicted probabilities of freedom from restenosis after treatment. *CAS*, Carotid artery stenting; *CEA*, carotid endarterectomy.

Restenosis probabilities are displayed in Table 4. The risk of restenosis was the same in the first year after both procedures, followed by a lower risk in the CAS group throughout the follow-up. (Fig 1) There was a statistically significant difference between the two groups; the P-value from the log-rank test was 0.045. The crude incidence rate of restenosis was 2.5/100 person-years in the CAS group and 4.2/100 person-years in the CEA group; the crude HR estimated by Cox regression was 1.80 (95% CI, 1.05–3.10; P = 0.030). Fig 2 shows the observed and predicted probabilities of being free from restenosis by treatment. The adjusted HR hardly differed from the crude being 1.85 (95% CI, 0.95–3.60; P = 0.070). The final model, which contained all important covariates (either being significant in the model or their omission would have changed the effect size more than 10%), included smoking, hypertension, diabetes mellitus, level of calcification and echogenicity, and location of the lesions, besides the type of treatment.

**Table 4:** Restenosis probability.

Restenosis probability		0	1 year	2 years	3 years	5 years	11 years
CEA	%	0	10.4	16.6	18.4	22.3	33.7
	No. at risk	116	104	93	89	76	41
	95% CI	0	6.07–17.64	10.90–24.72	12.40–26.81	15.64–31.22	25.11–44.24
CAS	%	0	11.4	11.4	12.4	14.7	17.2
	No. at risk	116	101	94	80	62	15
	95% CI	0	6.79–18.84	6.79–18.84	7.54–20.07	15.64–31.22	25.11–44.24

*CAS*, Carotid artery stenting; *CEA*, carotid endarterectomy; *CI*, confidence interval.

All patients received an antiaggregant, 60 of whom were on long-term dual antiplatelet therapy. Cilostazol therapy was noted in 13 patients (11.1%). Ninety-three patients (79.5%) received statin therapy, 51 of whom were on high-intensity therapy. Other lipid-lowering medications (fibrates and ezetimibe) were prescribed to 14 patients (12.0%).

## Discussion

The outcomes of CEA and CAS performed for ICA stenosis on the same patient have been previously investigated in four retrospective studies [19–21]. De Borst et al. compared the restenosis rates of 63 patients who underwent CEA first, followed by CAS. The median follow-up time was 54.4±39.5 months for CEA and 28.7±16.9 months for CAS. The ≥50% restenosis rates were not significantly different between CEA and CAS and were 10%, 19%, and 24% for CEA and 23%, 31%, and 34% for CAS at 1, 2, and 3 years, respectively [19]. Martelli et al. revealed a ≥50% restenosis rate of 31% for CEA, with a median follow-up time of 67.3±51.6 months, and 21% for CAS, with a median follow-up time of 24.6±16.9 months. In their study, the restenosis-free survival rate was 85% after CEA and 66% after CAS. As the definition of restenosis-free survival rate cannot be found in the article by Martelli et al. and the loss to follow-up is not reported either, survival rates mentioned by this group should be taken with a grain of salt [20]. Ciccone et al. evaluated 45 patients treated with CEA on one side and CAS on the contralateral side and did not observe restenosis ≥50% on either side of the patients [21]. Xu et al. carried out simultaneous CEA and CAS on eight patients and did not detect restenosis during the 6-month follow-up [22].

In our study, the risk of ≥50% restenosis after both procedures were the same in the first year, then it was significantly higher in the CEA group throughout the follow-up. Regarding CEA, we noted similar restenosis rates to those documented by De Borst et al. [19] but approximately a 10% better 5-year restenosis rate compared to that for Martelli et al. [20]. In the latter study, the majority of CEAs were performed with direct suturing, which is known to have higher restenosis rates compared to that in patching and EEA [36]. Regarding CAS, De Borst et al. published significantly worse restenosis rates at 1, 2, and 3 years compared to our results, but almost 20% of their CAS lesions were postsurgical restenoses [19], which has been previously identified as an independent risk factor for restenosis after CAS [20, 37].

According to a recently updated Cochrane meta-analysis, RCTs comparing restenosis rates of CEA and CAS showed a significantly higher risk for ≥50% restenosis after CAS (OR, 2.00; 95% CI, 1.12–3.60; P = 0.02; I^2^ = 44%); however, in terms of severe (≥70%) restenosis, no significant difference was observed in the pooled data (OR, 1.26; 95% CI, 0.79–2.00; P = 0.33; I^2^ = 58%) [38]. In the present study, most of the restenoses were 50–69% on the stented side and ≥70% on the operated side. Even after adjustment for differences in the two groups, a 1.86 HR was estimated favoring CAS with respect to restenosis. A possible explanation for our better patency rates for CAS could be that, in most of the RCTs, patients had a similar prognosis at baseline due to randomization, while our patients had different lesion characteristics on the two sides. Another explanation could be that differences in restenosis rates in our study were accentuated in the later stages of the follow-up, and none of the RCTs had as long a follow-up as our study.

Two main processes are playing roles in the pathomechanism of restenosis. One of them is neointimal hyperplasia, which occurs at the early stages, and the other one is neoatherosclerosis, which does not develop until 2 to 3 years after the intervention [39]. No significant difference was noted in terms of restenosis rate at 1 year between CEA and CAS, and almost half of the restenoses were found after 2 years in the CEA group. In our opinion, this underlies the importance of the duration of atherosclerotic risk factors, such as the average durations of hypertension, hyperlipidemia, and diabetes mellitus were significantly longer at the end of the follow-up on the side where CEA was performed.

One of the limitations is the retrospective nature of our study. Other limitations are the inevitable selection bias, as guidelines suggest different interventional techniques for different types of lesions. Although we adjusted the most important risk factors, residual confounding could not be excluded.

In conclusion, late restenosis is more frequent after CEA in patients undergoing both CEA and CAS on different sides for atherosclerotic bilateral carotid artery stenosis.

## Supporting information

S1 Table Evaluated parameters. (XLS)
